# Do lipids influence the allergic sensitization process?

**DOI:** 10.1016/j.jaci.2014.04.015

**Published:** 2014-09

**Authors:** Merima Bublin, Thomas Eiwegger, Heimo Breiteneder

**Affiliations:** a Department of Pathophysiology and Allergy Research, Medical University of Vienna, Vienna, Austria; Department of Pediatrics and Adolescent Medicine, Medical University of Vienna, Vienna, Austria

**Keywords:** Allergy, food allergens, immunomodulatory lipids, lipid-binding allergens, microbial lipids, pollen allergens, pollen-associated lipid mediators, pollen lipids, DC, Dendritic cell, iNKT, Invariant natural killer T, MD-2, Myeloid differentiation factor 2, MoDC, Monocyte-derived immature dendritic cell, nsLTP, Nonspecific lipid transfer protein, PALM, Pollen-associated lipid mediator, PPE_1_, Phytoprostane E_1_, TLR, Toll-like receptor

## Abstract

Allergic sensitization is a multifactorial process that is not only influenced by the allergen and its biological function *per se* but also by other small molecular compounds, such as lipids, that are directly bound as ligands by the allergen or are present in the allergen source. Several members of major allergen families bind lipid ligands through hydrophobic cavities or electrostatic or hydrophobic interactions. These allergens include certain seed storage proteins, Bet v 1–like and nonspecific lipid transfer proteins from pollens and fruits, certain inhalant allergens from house dust mites and cockroaches, and lipocalins. Lipids from the pollen coat and furry animals and the so-called pollen-associated lipid mediators are codelivered with the allergens and can modulate the immune responses of predisposed subjects by interacting with the innate immune system and invariant natural killer T cells. In addition, lipids originating from bacterial members of the pollen microbiome contribute to the outcome of the sensitization process. Dietary lipids act as adjuvants and might skew the immune response toward a T_H_2-dominated phenotype. In addition, the association with lipids protects food allergens from gastrointestinal degradation and facilitates their uptake by intestinal cells. These findings will have a major influence on how allergic sensitization will be viewed and studied in the future.

Lipids are hydrophobic or amphipathic small molecules. The International Lipid Classification and Nomenclature Committee organizes lipids into 8 well-defined categories^1^ among which certain members of the fatty acids, glycerolipids, glycerophospholipids, sphingolipids, and saccharolipids have been experimentally shown to be involved in allergic sensitization. Binding or colocalization of immunostimulatory or immunomodulatory lipids could significantly contribute to the allergenicity of proteins. Although conventional activation of pathogen recognition receptors is considered to result in T_H_1-dominated responses, it becomes clear that Toll-like receptor (TLR)–, NOD-like receptor–, or C-type lectin receptor–triggering stimuli are also able to induce T_H_2 responses.^2^ Sensitization by means of inhalation of low levels of LPS in concert with the respective allergen results in T_H_2-type lung inflammation through TLR4, whereas high amounts of LPS induces a T_H_1 response.^3^, ^4^ Lipid binding is a common characteristic observed for members of several protein families that include allergens, such as Bet v 1–like proteins, nonspecific lipid transfer proteins (nsLTPs), 2S albumins, secretoglobulins, lipocalins, oleosins, and mite group 2, 5, and 7 proteins (Table I).^5^, ^6^, ^7^, ^8^, ^9^, ^10^, ^11^, ^12^, ^13^, ^14^, ^15^, ^16^, ^17^, ^18^, ^19^, ^20^, ^21^, ^22^, ^23^, ^24^, ^25^, ^26^, ^27^, ^28^ These proteins contain hydrophobic binding sites for lipid ligands, and immunomodulation by such binding partners leads to T_H_2-enhancing actions.

**Table I:** Lipid-binding allergens

Protein family	Allergen	Source	Mode of lipid binding and ligands	Effects and mechanism of action
Bet v 1 like	Bet v 1	Birch pollen *(Betula verrucosa)*	Binds and transports diverse hydrophobic ligands in a large hydrophobic cavity^5^, ^6^, ^7^	Binding and permeabilization of membranes^5^
	Ara h 8	Peanut *(Arachis hypogaea)*	Associated with lipid fraction of raw and roasted peanuts	Ara h 8 isolated from roasted peanuts showed higher thermal and proteolytic stability^18^
	Mal d 1	Apple *(Malus domestica)*	Penetrates phospatidylcholine vesicles	Interaction with phospatidylcholine induced enhanced basophil activation^19^
MD-2–related domain	Der p 2	House dust mite *(Dermatophagoides pteronyssinus)*	Bind LPS because of structural similarity to MD-2, the LPS-binding component of the TLR4 complex^9^	Der p 2 promotes TLR4 signaling and induces a robust airway T_H_2 inflammation in wild-type but not TLR4-deficient mice^8^
	Der f 2	House dust mite *(Dermatophagoides farinae)*		
Group 5/21 mite allergen	Der p 5	House dust mite	Possibly binds hydrophobic ligands in a large hydrophobic cavity^10^	Unknown
Group 7 mite allergen	Der p 7	House dust mite	Binds the bacterial lipopeptide polymyxin B^11^	Unknown
Group 1 cockroach allergen	Bla g 1	Cockroach *(Blattella germanica)*	Binds different lipids, such as palmitic, oleic, and stearic acids, through hydrophobic cavity^12^	Unknown
Fel d 1 family	Fel d 1	Cat *(Felis domesticus)*	Binds LPS	Enhances LTA/TLR2 and LPS/TLR4 signaling in both a transfected cell model and in primary macrophage-like cells^13^
Lipocalin	Can f 6	Dog *(Canis familiaris)*	Binds LPS	Enhances LPS/TLR4 signaling in both a transfected cell model and in primary macrophage-like cells^13^
	Bos d 5	β-Lactoglobulin from cow's milk	Inserts into the lipid bilayer^24^	Interaction with phospatidylcholine protects β-lactoglobulin from breakdown in an *in vitro* gastroduodenal environment^24^
Nonspecific lipid transfer protein	Par j 1	Parietaria *(Parietaria judaica)*	Binds LPS through C-terminal Par37 peptide	Inhibition of LPS-induced IL-6 and TNF-α in RAW264.7 cells, inhibition of LPS-induced INF-γ secretion in murine spleen cells and human PBMCs^27^
	Pru p 3	Peach *(Prunus persica)*	Tunnel-like lipophilic cavity capable of binding different lipid ligands^28^	Internalization by Caco 2 cells through an endocytic pathway involving lipid rafts and caveolar endocytosis accompanied by expression of T_H_2 cytokines^14^
2S albumin	Ber e 1	Brazil nut *(Bertholletia excelsa)*	Potentially lipid-binding hydrophobic cavity	rBer e 1 coadministrated with lipid fractions isolated from Brazil nuts induced an IgE and IgG_1_ antibody response^15^ Ber e 1/lipid-stimulated murine and human CD1d-restricted iNKT cells produced IL-4 but not IFN-γ^16^
	Sin a 1	Mustard *(Sinapis alba)*	Interacts with acidic phospholipid vesicles	Permeabilization of lipid bilayer^17^
Oleosins	Ara h 10 Ara h 11	Peanut *(Arachis hypogaea)*^20^	Structural proteins of oil bodies	Unknown
	Cor a 12 Cor a 13	Hazelnut *(Corylus avellana)*^20^		
	Ses i 4 Ses i 5	Sesame *(Sesamum indicum)*^21^		
Thiol protease	Gly m Bd30K	Soy *(Glycine max)*	Oil body–associated allergen	Enhanced absorption of intact and fragments of Gly m Bd 30K in mice through a fat carrier–mediated transport^22^
Vicilin	Gly m 5	Soy *(Glycine max)*	Associates with oil bodies by forming a complex with Gly m Bd 30K through a disulfide bond^23^	Unknown
C-type lysozyme	Bos d 4	α-Lactalbumin from cow's milk	Interacts with phospatidylcholine	Protective effect of phospatidylcholine against gastrointestinal digestion^25^

Allergen-bound ligands are not the only source of immunomodulatory lipids. Lipids are present in high concentrations in pollen coats and in matrices of plant and animal foods. Lipids protect pollen grains against UV light damage or attack by pathogens, and they play a key role in pollen-pistil interactions.^29^ Microbial contaminations are additional sources of immunomodulatory lipids. Pollen is never sterile. Thus far, the study of pollen microbiomes has been limited to analyzing bacterial communities acquired by honey bees through a horizontal transmission from pollen and flower nectars.^30^ The sensitization process is modulated by several key players, of which lipids are clearly one (Table II).^26^, ^31^, ^32^, ^33^, ^34^, ^35^, ^36^, ^37^, ^38^, ^39^, ^40^, ^41^, ^42^, ^43^, ^44^ Hence future studies of the mechanisms of sensitization are well advised to include lipids in their experimental setups.

**Table II:** Overview of lipid mediators and their mechanisms of action

Lipids	Source	Experimental setting	Effects	Mechanism of action	References
Microbial lipids in pollen grains	Extract of gram-positive bacteria, *Bacillus cereus* and *Bacillus subtilis*, from timothy *(Phleum pratense)* pollen grains	Human immature DCs	↑ Maturation markers ↑ IL-6, IL12 p40, and TNF-α in allergic donors	Adjuvant activity by enhancing maturation of DCs and induction of T_H_1-, T_H_2-, and T_H_17-mediated allergic inflammation	Heydenreich et al^31^
		DC/naive T-cell coculture	Allergen-specific proliferation and ↑ production of IL-4, IL-10, IL-13, IL-17, IL-22, and IFN-γ		
	Ryegrass *(Lolium perenne)* pollen extracts with LPS content >5000 EU/mL containing bacterial lipopeptides and CpG motifs	Human PBMCs	↑ IFN-γ^+^ and IL-4^+^ cells; ↓ CD4^+^ Foxp3^hi^ Treg cells ↑ IFN-γ, TNF-α ,IL-5, IL-10	T_H_1- and T_H_2-biased proinflammatory response with reduction in Treg cell numbers	Mittag et al^32^
		HEK293 cells expressing TLRs	Activation of TLR4, TLR2, and TLR9		
	Glycolipid α-glycuronosylceramides	BALB/c mice immunized with OVA and α-galactosylceramide	↑ Airway hyperreactivity ↑ Airway eosinophils ↑ IgE and T_H_2 cytokines	α-Glycuronosylceramides act as adjuvants binding to CD1d and activating Vα14 iNKT cells	Kim at al^43^
		Mouse *in vivo* studies	Local activation of natural killer T cells and release of IL-4 and IFN-γ followed by activation of DCs and allergen-specific CD4^+^ T_H_2 cells		Scanlon et al^44^
Pollen lipids	Phospatidylcholine and phospatidylethanolamine from cypress pollen grains (*Cupressus arizonica* and *Cupressus sempervirens*)	Autologous DCs and T-cell lines from patients with cypress pollen allergy	Proliferation of pollen lipid–specific T_H_2 cells secreting IL-4 and IFN-γ ↑ Lipid-specific IgE	CD1a- and CD1d-mediated recognition of pollen lipids by T cells	Agea et al^33^
	Total lipid fractions from olive pollen *(Olea europaea)*	PBMCs from healthy donors	↑ IL-4 and IFN-γ ↑ Expression of CD1d on DCs ↓ Expression of CD1a	Activation of iNKT cells in a CD1d-dependent way through PPARγ	Abos-Gracia et al^34^
PALMs	Aqueous and lipid extracts from grass *(Phleum pratense)* and birch *(Betula alba)* pollen	Human neutrophils and eosinophils	Attraction and activation of neutrophils and eosinophils ↑ CD11b	Chemoattractant activity, which generates a T_H_2-promoting micromilieu	Plotz et al^35^ Traidl-Hoffmann et al^36^
	Aqueous birch *(Betula alba)* pollen extract	Human LPS-maturated MoDCs	Inhibition of LPS-induced production of the T_H_1-attracting chemokines CXCL10 and CCL5 ↑ Release of CCL22		Mariani et al^38^
	Phytoprostanes E_1_	Human LPS-maturated MoDCs	Inhibition of LPS-induced IL-12 p70 through inhibition of PPARγ signaling and consecutive T_H_2 polarization	Polarization of naive T cells to T_H_2 type by inhibiting DCs to produce T_H_1 cytokine IL12 p70 or IL-6	Gilles et al^37^ Traidl-Hoffmann et al^39^
	Phytoprostanes E_1_	slanDCs	Inhibition of LPS-induced IL-12 p70 and IL-6		Gilles et al^40^
	Low-molecular-weight nonprotein factors from *Betula alba*	LPS-stimulated-slanDCs	Inhibition of LPS-induced surface expression of T-cell costimulatory markers CD80 and CD40 and chemokine receptor CCR7	Modulation of a native human DC subset on the level of cytokine production, costimulation, and ensuing T-cell response	Gilles et al^40^
Dietary MCTs	Peanut butter	Oral sensitization of naive C3H/HeJ mice with peanut butter proteins with MCTs	IgG-dependent anaphylaxis after systemic challenge and IgE-dependent anaphylaxis after oral challenge; Promote T_H_2 cytokine responses in splenocytes	Stimulation of T_H_2 responses by affecting antigen absorption and availability	Li et al^41^
Sphingolipids	Cow's milk	PBMCs from children with cow's milk allergy or eosinophilic esophagitis	Sphingolipid-dependent iNKT cell proliferation and secretion of T_H_2 cytokines IL-4, IL-5, and IL-13	Activation of peripheral blood iNKT cells to produce T_H_2 cytokines and eosinophil-mediated inflammation	Jyonouchi et al^26^, ^42^

*Foxp3*, Forkhead box protein 3; *LTA*, lipoteichoic acid; *MCT*, medium-chain triglyceride; *OVA*, ovalbumin; *PPARγ*, peroxisome proliferator-activated receptor γ; *slanDC*, 6-sulfo LacNac^+^ dendritic cell; *Treg*, regulatory T.

## Interaction of lipids and inhalant allergens

Exposure to pollens as a major source of inhalant allergens can induce the production of specific IgE in predisposed subjects and thus represents a risk for development of asthma/rhinitis and eczema.

The pollen coat contains a large range of lipids that are required for pollen hydration, germination, and penetration of the stigma by the pollen tube.^45^ These pollen coat lipids might possess immunomodulatory characteristics and could thus influence the antigenic properties of pollen proteins. Pollen proteins with lipid-binding activity might even pick up such lipids and deliver them to the immune system. Bashir et al^45^ performed a lipidomic analysis of lipids from pollen of 22 species (14 tree species, 6 grass species, and 2 weed species) to establish a database of lipid antigens as new candidate molecules involved in allergy.

### Lipid ligands of pollen allergens

The structure of the major birch pollen allergen Bet v 1 consists of a 7-stranded anti-parallel β-sheet that wraps around a long C-terminal α-helix while 2 consecutive short α-helices connect the β1- and β2-strands.^46^ A large hydrophobic cavity penetrates the entire protein, suggesting that Bet v 1 might function as a carrier or storage protein for hydrophobic ligands (Table I). The isoform Bet v 1.0101 was shown to bind the fluorescent probe 8-anilino-1-naphthalenesulfonic acid, which could be displaced by fatty acids, flavonoids, and cytokinins, indicating a possible promiscuity in ligand binding.^5^ Two deoxycholate molecules found in the hydrophobic cavity of isoform Bet v 1.0107 (previous names: Bet v 1.1001 and Bet v 1l), although an artifact from the preparation of the recombinant protein, suggest that Bet v 1 might also bind plant hormones of the brassinosteroid class.^6^ The structure of 2 Bet v 1 isoforms in complex with the flavonoid naringenin and the cytokinin kinetin became available recently.^47^

Bet v 1 undergoes structural rearrangements when binding to phospholipid vesicles,^48^ thus very likely releasing any bound natural ligand. Very recently, the natural ligand of Bet v 1 was identified as the glycosylated flavonol quercetin-3-*O*-sophoroside, and it was speculated that this ligand might affect the allergic sensitization process when released after contact with lipids.^49^ In a physiologic setting, these lipids are present in the pollen coat or the exudate of the stigma, or during the pathogenesis of birch pollinosis, these lipids are present in the membranes of nasal or conjunctival epithelial cells. Contact with lipids seems to change the conformation of Bet v 1, leading to the release of its natural ligand to perform its possible role in signal transduction.^7^ It is not known whether Bet v 1, after releasing its ligand, binds to any other lipids. If this were the case, one might speculate that lipids transported by Bet v 1 could also originate from bacterial contaminations of the pollen.

### Microbial lipids

Although bacteria associated with roots and leaf surfaces are being studied extensively,^50^ very little is known about pollen microbiomes. Supernatants of gram-positive *Bacillus cereus* and *Bacillus subtilis* present on timothy pollen grains induced the maturation of monocyte-derived immature dendritic cells (MoDCs) from donors with grass pollen allergy.^31^ In cocultures of autologous CD4^+^ T cells and dendritic cells (DCs) treated with grass pollen extracts, addition of supernatants of homogenized gram-positive bacteria or LPS led to an enhanced secretion of T_H_1, T_H_2, and T_H_17 cytokines (Table II). In experiments with MoDCs from nonallergic donors, little or no T_H_2 cytokines could be detected. Mittag et al^32^ found that ryegrass pollen extracts had highly variable LPS contents. Stimulation of TLR-transfected cell lines revealed that pollen with a high LPS content also contained a TLR2 ligand indicative of bacterial lipopeptides. Extracts with low or high LPS content additionally contained a TLR9 ligand, indicating the presence of CpG motifs from bacterial DNA. Interestingly, the coexposure to allergen and proinflammatory microbial stimuli did not increase the T_H_1 bias in nonatopic subjects or convert an established T_H_2 response into a T_H_1 response in allergic subjects. Both T_H_1- and T_H_2-biased responses were exacerbated, whereas CD4^+^ forkhead box protein 3–high regulatory T-cell induction was decreased. Obviously, pollen contains additional factors that suppress the effect of LPS and enhance the sensitization process. When murine bone marrow–derived DCs were stimulated with pollen extracts or pollen grains from Japanese cedar, Japanese cypress, birch, ragweed, and Kentucky bluegrass in the presence of LPS, various degrees of inhibition of LPS-induced IL-12 and TNF-α production were observed.^51^

Pollen allergens can also actively bind LPS. Par j 1, an allergenic nsLTP from *Parietaria* species pollen, exists in 2 isoforms. Par j 1.0101 has a 37-amino-acid residue extension (Par37) at its C-terminus compared with the shorter Par j 1.0201. Par37 possesses features of peptides involved in host defense. When used as a synthetic peptide, Par37 showed LPS-binding activity and inhibited LPS-induced IL-6 and TNF-α expression at the mRNA and protein levels in the mouse macrophage cell line RAW264.7.^52^ In addition, Par37 reduced the ability of human PBMCs to secrete INF-γ after exposure to LPS, illustrating its ability to modulate cytokine production of antigen-presenting cells (Table I).

### Pollen lipids

Phospatidylcholine and phospatidylethanolamine present on the surface of cypress pollen are relevant for the capture of pollen grains by mucosal DCs through CD1 molecules, and they also stimulate the proliferation of T cells from subjects with cypress pollen allergy.^33^ The αβ T-cell clones isolated from subjects with cypress pollen allergy showed a tendency to produce both IFN-γ and IL-4, suggesting their involvement in inflammatory and allergic responses (Table II). Peripheral blood– and nasal mucosa–associated γδ T cells from subjects with cypress pollen allergy, but not healthy control subjects, were found to recognize pollen-derived phosphatidylethanolamine in a CD1d-restricted fashion.^53^ Proliferating clones secreted both T_H_1- and T_H_2-type cytokines and drove IgE production *in vitro* and *in vivo*.

Invariant natural killer T (iNKT) cells are CD1d-restricted T lymphocytes that use a very limited T-cell receptor repertoire to recognize self-lipids and foreign lipids.^54^ iNKT cells are cytotoxic and able to secrete both T_H_1 and T_H_2 cytokines. A recent study analyzed the general ability of olive pollen lipids to activate DCs and stimulate iNKT cells, both of which were derived from healthy donors (Table II).^34^ Polar lipids, diacylglycerols, free fatty acids, and triacylglycerols isolated from olive pollen grains upregulated CD1d on DCs, which then activated iNKT cells in cocultures. No cytokine secretion measurements were reported, and PBMCs, monocytes, or iNKT cells from subjects with olive pollen allergy were not included in this study.

### Pollen-associated lipid mediators

Pollen grains contain pollen-associated lipid mediators (PALMs), eicosanoid-like molecules that are rapidly released in a humid environment and that favor T_H_2-dominated allergic immune responses.^55^ PALMs can be divided into the leukotriene B_4_–like group that induces chemotaxis and activation of human neutrophils and eosinophils^35^, ^36^ and the phytoprostane group that inhibits the production of IL-12p70 in human DCs through blocking of nuclear factor κB nuclear translocation and a mechanism involving the nuclear receptor peroxisome proliferator-activated receptor γ.^37^ Aqueous pollen extracts from birch pollen induced CXCR4 upregulation in immature DCs with concomitant downregulation of CCR1 and CCR5 and modulated LPS-induced maturation of monocyte-derived DCs through reducing levels of the T_H_1-related chemokines CXCL10 and CCL5 and downregulating CCL22, a T_H_2 chemokine (Table II).^38^ Pollen-derived phytoprostane E_1_ (PPE_1_), which represents the most prominent group of phytoprostanes, shows homology to prostaglandin E_2_ and is responsible for these T_H_2-favoring effects.^39^ PALMs were identified in aqueous extracts from tree, weed, and grass pollen.^56^ 6-Sulfo LacNac^+^ DCs, the most abundant native DC population in human peripheral blood with a constitutively high potency to induce T_H_1 responses, were shown to be susceptible to the T_H_2-polarizing effect of PPE_1_ from birch pollen.^40^ PPE_1_ inhibited the LPS-induced IL-12 p70 production and the secretion of IL-6.

### Lipid-binding allergens from house dust mites and cockroaches

The house dust mite allergens Der p 2 from *Dermatophagoides pteronyssinus* and Der f 2 from *Dermatophagoides farinae* possess a myeloid differentiation factor 2 (MD-2)–related lipid recognition domain that is implicated in lipid recognition (Table I). MD-2 is the LPS-binding component of the TLR4 signaling complex.^57^ Trompette et al^8^ showed that Der p 2 in the presence of LPS promoted TLR4 signaling and that Der p 2 along with very low LPS concentrations induced a robust airway T_H_2 inflammation in wild-type but not TLR4-deficient mice. Der p 2 was also able to replace MD-2 function in TLR4 signaling in the absence of MD-2. This might facilitate antigen presentation and effector cell activation and restore TLR4 signaling in the bronchial epithelium because bronchial epithelial cells express TLR4 but not MD-2.^58^ Although this study elucidates the mechanism underlying the allergenicity of Der p 2, it does not explain why only particular subjects become allergic after exposure to this allergen. Der f 2 was shown to bind LPS with an affinity comparable with that reported for MD-2.^9^

Der p 5, which is a 3-helical bundle, has a tendency to form dimers because of the presence of a valine zipper (Table I).^10^ The large hydrophobic cavity observed in the Der p 5 dimer was occupied by methylpentanediol molecules present during crystallization. This suggests that Der p 5 has the potential to bind hydrophobic ligands. The fold of Der p 7 resembles that of the LPS-binding protein, which interacts with TLR4 after binding LPS and other bacterially derived lipid ligands.^11^ However, Der p 7 did not bind LPS but bound the bacterial lipopeptide polymyxin B with weak affinity, as was shown for Der f 7 as well.^59^ Interestingly, a *D pteronyssinus* extract induced signaling through TLR2 and TLR4 in the mouse alveolar macrophage cell line MH-S.^60^ The actual immune-stimulatory, proallergenic binding partners still need to be defined in detail.

The cockroach allergen Bla g 1 consists of 4 to 14 repeats of approximately 100 amino acid residues each, which form a novel fold of 6 helices.^12^ Two repeats encapsulate a large and nearly spherical hydrophobic cavity, which holds, depending on the allergen origin, palmitic, oleic, or stearic acids, indicating a function associated with nonspecific transport of lipid molecules. The lipids associated with Bla g 1 are known to activate TLR2 and TLR4 and can subsequently skew the adaptive immune response toward allergy.^61^ In the context of Bla g 1, the immunomodulatory capacities of these lipids are awaiting experimental confirmation.

### Lipid-binding inhalant mammalian allergens

The cat dander protein Fel d 1 is a major inhalant allergen and associated with allergic reactions at exposure among sensitized patients. Fel d 1, a secretoglobulin, is not a homolog of the TLR4-assciated LPS-binding protein MD-2 and hence cannot mimic its biological function, as does Der p 2. Herre et al^13^ showed that Fel d 1 enhanced lipoteichoic acid–induced TLR2 signaling approximately 15-fold and LPS-induced TLR4 signaling approximately 2.5-fold in transiently transfected HEK293 cells. In primary human PBMCs Fel d 1 also enhanced LPS-induced TNF-α production. The mechanism for TLR enhancement of signaling involves the formation of complexes between the bacterial lipids and the allergen and a direct transfer to the TLRs on the cell surface. In the same study the dog allergen Can f 6, a member of the lipocalin family, was shown to have lipid-complexing properties very similar to those of Fel d 1. Lipocalins form an 8-stranded β-barrel structure with a hydrophobic cavity to which hydrophobic ligands can bind. Most of the important mammal-derived respiratory allergens, including those from cat, dog, horse, and mouse, belong to the lipocalin family.^62^ However, a recent study by Parvainen et al^63^ found that near to endotoxin-free lipocalin allergens had no effect on the activation or cytokine production of human MoDCs. This might indicate that the presence of lipid ligands is necessary for these allergens to be able to skew the immune response toward a T_H_2 phenotype.

## Interaction of lipids and food allergens

An increasing number of studies indicate that interaction with lipids can fundamentally alter the ability of food allergens to reach the sites of active immune sampling in the gut-associated lymphoid tissue. Lipids can facilitate the passage of an allergen through the intestinal epithelial barrier, alter their degradation within the gastrointestinal tract, or both and thus affect the potential allergenicity of a protein. In addition, recent studies have demonstrated that some dietary lipids act as adjuvants, activating innate immunity followed by enhanced allergen-specific immune responses when used in combination with a specific allergen.

Many plant and animal food allergens occur as allergen-lipid complexes. Allergens can bind lipids in hydrophobic cavities either through electrostatic interactions or through less well-defined association with lipids because of the presence of hydrophobic patches that lie close to the protein surface. In addition to naturally occurring protein-lipid complexes, such complexes can also be generated during food processing by roasting or emulsion formation or during food storage because of lipid oxidation. Animal fats and vegetable oils are sources of lipids containing triacylglycerols (95%), phospholipids (approximately 4.5%), and cholesteryl esters. During digestion, bile provides additional lipids, such as phosphatidylcholine, bile acids, and unesterified cholesterol, as major species. Protein-lipid complexes can thus be induced in the duodenum, where dietary components are mixed together, with bile and peristalsis as major contributing factors for the emulsification.^64^

### Plant food allergens

nsLTPs, members of the prolamin superfamily, have been identified as allergens in a wide range of plant foods, including fruits, vegetables, peanuts, tree nuts, and cereals.^65^ They are bundles of α-helices stabilized by 4 or 5 disulfide bounds. nsLTPs possess a flexible tunnel-like lipophilic cavity that is capable of binding a wide variety of lipid ligands, such as phospholipids, different fatty acids, palmitoyl-CoA, or prostaglandin B_2_. It has been speculated that the antimicrobial activity of nsLTPs could result from the interaction and permeabilization of biological membranes.^66^ Tordesillas et al^14^ showed that the nsLTP from peach, Pru p 3, was internalized by Caco 2 cells through an endocytic pathway involving lipid rafts and caveolar endocytosis. The authors further reported that the lower transport rate of a hypoallergenic peach nsLTP was associated with a significantly lower expression of T_H_2-related cytokines compared with Pru p 3 (Table I). The presence of gastric phospatidylcholine in an *in vitro* digestion assay had a protective effect on the grape nsLTP.^67^ The breakdown of the protein by duodenal enzymes was slowed down, resulting in a slightly higher ability of the allergen to induce basophil histamine release and to elicit skin reactions in 4 patients with grape allergy.

The 2S albumin from Brazil nut, Ber e 1, has a potentially lipid-binding hydrophobic cavity of the same size as nsLTPs.^68^, ^69^ It has been shown that Ber e 1 by itself was not sufficient to cause IgE or IgG production in mice.^15^ IgE and IgG_1_ antibody responses were induced only when recombinant Ber e 1 was coadministered with the total, sterol-rich, or polar lipid fractions isolated from Brazil nuts (Table I). The β-sitosterol and glycolipid-rich fractions of Brazil nut lipids had little effect on the sensitization process. This effect was partially dependent on iNKT cells because Ber e 1–specific IgE levels were lower in the absence of iNKT cells. Human T-cell lines containing more natural T cells produced more IL-4 in response to stimulation with Ber e 1 mixed with a fraction containing neutral and common phospholipids isolated from Brazil nut.^16^ Although the mode of lipid association with Ber e 1 was not exactly defined, the fact that a stoichiometry of protein/lipid (1:1) was sufficient to induce the observed responses suggests that Ber e 1 might accommodate lipids in its hydrophobic pocket. However, it remains to be elucidated whether the observed effect is induced by Ber e 1–lipid complexes or whether the type of lipid coadministered with Ber e 1 determines the antigen response because of an immunomodulating effect. For example, it has been shown that medium-chain but not long-chain triglycerides could promote allergic sensitization and anaphylaxis to coadministered peanut proteins in mice by affecting absorption and stimulating a T_H_2 response (Table II).^41^ In addition, 2S albumins, such as Sin a 1 from yellow mustard or different 2S albumin isoforms from sunflower seeds, were able to bind and associate with lipids.^17^, ^70^ Sin a 1 has also been shown to strongly interact with acidic phospholipid vesicles, perturbing the bilayer and causing the formation of leaky structures, although probably not through the action of a binding pocket, as is the case for members of the nsLTP family, but through their activity as efficient emulsifiers, as shown for SFA8/7 from sunflower.^70^

Proteins belonging to the Bet v 1–like family are also able to bind lipids and associate with lipid membranes (Table I).^71^ Food allergens of the Bet v 1–like family generally are susceptible to pepsin digestion and instable to thermal processing and usually provoke only mild allergic symptoms. A recent study on Ara h 8, the Bet v 1 homolog from peanut, showed that roasting of peanuts can dramatically increase not only its thermal stability but also protect the allergen against proteolysis by gastrointestinal enzymes.^18^ Ara h 8 isolated from roasted peanuts showed enhanced IgE reactivity and was also recognized by more sera from patients with peanut allergy compared with Ara h 8 isolated from raw peanuts. During storage or roasting, peanuts readily undergo lipid oxidation because of their high polyunsaturated fatty acid content. The formed lipid hydroperoxides or their breakdown products might attack proteins and induce formation of new protein-lipid complexes and also changes protein structures. In addition, Sancho et al^19^ demonstrated that phosphatidylcholine affected the secondary structure of Bet v 1–homologous allergens from apple, cherry, and hazelnut and also enhanced the ability of the digested allergens to induce histamine release. The fact that Ara h 8 could only be isolated from the lipid fraction of peanuts explains its absence in aqueous peanut extracts and the resulting false-negative diagnostic test results. However, isolated Ara h 8 sensitization is usually associated with tolerance or induction of typical mild symptoms to roasted peanuts.^72^, ^73^

Naturally occurring protein-lipid complexes are also present in the oil bodies of oil seeds. Oleosins, structural proteins of oil bodies, have been identified as allergens in peanut, sesame, soybean, hazelnut, and olive fruit (Table I).^20^, ^21^, ^74^ A highly conserved central hydrophobic domain of the protein penetrates the phospholipid layers, whereas the N- and C-terminal hydrophilic portions of the protein interact electrostatically with phospholipids on the surfaces of oil bodies.^75^ Data about the allergenic properties of oleosins are few. This is due to difficulties encountered during purification and handling because all membrane proteins lose their functions and fold once removed from the lipid bilayer and are usually irreversibly denaturated.

The soy allergen Gly m Bd 30K, a member of the thiol protease family, is another oil body–associated allergen. The association of Gly m Bd 30K with oil bodies was critical for the enhanced absorption of intact and digested fragments of Gly m Bd 30K in mice through a fat carrier–mediated transport.^22^ Gly m Bd 30K forms a complex through a disulfide bond with another important soy allergen, Gly m 5, which, together with Gly m 6, is associated with oil bodies isolated from soy.^23^ The association with oil bodies might protect these allergens from digestion and allow an increased cellular uptake through carrier-mediated transport, but further studies need to elucidate this aspect.

### Cow's milk allergens

The major mammalian milk allergen β-lactoglobulin belongs to the lipocalin protein family and is highly valued in the food industry for its emulsifying and emulsion-stabilizing properties. In dietary products β-lactoglobulin coexists with milk fat globular membranes, which consist of 27% phospholipids, including phosphatidylcholine, phosphatidylethanolamine, and sphingolipids. It was found that the adsorption of β-lactoglobulin onto lipid surfaces is mainly driven by strong electrostatic interactions of positively charged amino acid residues of the protein and negatively charged groups of phospholipids. These interactions induce β- to α-transitions in the protein through extensive disruption of the tertiary structure. Once the tightly packed β-lactoglobulin is disrupted, hydrophobic residues become exposed and readily available for insertion into the lipid bilayer.^76^ The importance of the intact structure and functionality for the immunogenic and sensitizing capacity of β-lactoglobulin was indicated by a recent study.^77^ Only the intact, but not the digested, β-lactoglobulin was able to induce IgG and IgE antibody responses in a Brown Norway rat model. Furthermore, β-lactoglobulin was shown to be protected from breakdown in an *in vitro* gastroduodenal environment, which is in marked contrast to the susceptibility of the protein to duodenal enzymes in the absence of the physiologic surfactant phospatidylcholine (Table I). Inhibition of β-lactoglobulin digestion by phosphatidylcholine resulted in increased allergenic activity of β-lactoglobulin, as determined by using skin prick tests.^24^ Phospatidylcholine was also found to have a protective effect on digestion of another milk allergen, namely α-lactalbumin.^25^

On the other hand, there is evidence that lipids present in milk *per se* might promote a T_H_2-skewed environment in predisposed subjects by inducing iNKT cells to secrete T_H_2 cytokines. Cow's milk, but not hen's egg, sphingolipids are capable of stimulating iNKT cells in a CD1d-dependent fashion and induce their expansion and the release of IL-4, IL-5, and IL-13. This effect is more pronounced in patients with cow's milk allergy (Table II).^42^ The relevance of this observation in context with the peak prevalence of milk allergy within the first years of life remains to be investigated. The same effects were seen in patients with eosinophilic esophagitis. Such patients display a lower frequency of iNKT cells in the periphery, whereas higher percentages of iNKT cells are observed at the site of inflammation. This indirectly suggests activation of iNKT cells to induce T_H_2-mediated inflammation through cow's milk–derived sphingolipids.^26^

## Concluding remarks

Allergens are never delivered to the immune system in a pure form. They are generally associated with allergen sources, such as pollen grains, food matrices, or fecal particles. These sources contain a variety of immunomodulatory components, among which lipids play a major role. Furthermore, bacterial contaminations contribute to the mixture of compounds that accompany the allergens. Our understanding of allergen-associated lipids in the generation of allergic sensitization is still at its beginning. The data summarized here indicate that lipids influence the early stages of allergic sensitization by interacting with several components of the innate immune system. On the basis of currently available data, TLR4- and TLR2-dependent mechanisms, either through conventional binding or interaction with the signaling pathway, and CD1d-restricted mechanisms are the most relevant. However, alternate means of T_H_2 induction, such as through the carbohydrate portion of glycolipids that result in T_H_2-type responses, seem likely and await further investigation.

Moreover, lipids can protect allergens against proteolysis and enhance their uptake by intestinal epithelial cells. Interestingly, the Brazil nut allergen Ber e 1 and lipocalins derive their allergenicity from the presence of lipids. It is tempting to speculate that the allergenicity of other lipid-binding proteins relies, to a certain extent, on the presence of lipids. The contribution of each of the allergen-associated lipids will have to be clearly determined within the complex mixture of immunomodulatory molecules. Hence future research will have to focus on identifying these lipids, characterizing their interaction with allergens, and analyzing the combined effects on cells of the immune system.
